# Family Aggregation of Human T-Lymphotropic Virus 1-Associated Diseases: A Systematic Review

**DOI:** 10.3389/fmicb.2016.01674

**Published:** 2016-10-28

**Authors:** Carolina Alvarez, Eduardo Gotuzzo, Anne-Mieke Vandamme, Kristien Verdonck

**Affiliations:** ^1^Instituto de Medicina Tropical Alexander von Humboldt, Universidad Peruana Cayetano HerediaLima, Peru; ^2^Department of Microbiology and Immunology, Clinical and Epidemiological Virology, Rega Institute for Medical Research, KU Leuven—University of LeuvenLeuven, Belgium; ^3^Departamento de Enfermedades Infecciosas, Tropicales y Dermatológicas, Hospital Cayetano HerediaLima, Peru; ^4^Center for Global Health and Tropical Medicine, Unidade de Microbiologia, Instituto de Higiene e Medicina Tropical, Universidade Nova de LisboaLisbon, Portugal; ^5^Department of Public Health, Institute of Tropical Medicine AntwerpAntwerp, Belgium

**Keywords:** human T-lymphotropic virus 1, tropical spastic paraparesis, adult T-cell leukemia-lymphoma, uveitis, family research, systematic review

## Abstract

Human T-lymphotropic virus 1 (HTLV-1) is a retrovirus that produces a persistent infection. Two transmission routes (from mother to child and via sexual intercourse) favor familial clustering of HTLV-1. It is yet unknown why most HTLV-1 carriers remain asymptomatic while about 10% of them develop complications. HTLV-1 associated diseases were originally described as sporadic entities, but familial presentations have been reported. To explore what is known about family aggregation of HTLV-1-associated diseases we undertook a systematic review. We aimed at answering whether, when, and where family aggregation of HTLV-1-associated diseases was reported, which relatives were affected and which hypotheses were proposed to explain aggregation. We searched MEDLINE, abstract books of HTLV conferences and reference lists of selected papers. Search terms used referred to HTLV-1 infection, and HTLV-1-associated diseases, and family studies. HTLV-1-associated diseases considered are adult T-cell leukemia/lymphoma (ATLL), HTLV-1-associated myelopathy/tropical spastic paraparesis (HAM/TSP), HTLV-1-associated uveitis, and infective dermatitis. Seventy-four records reported HTLV-1-associated diseases in more than one member of the same family and were included. Most reports came from HTLV-1-endemic countries, mainly Japan (*n* = 30) and Brazil (*n* = 10). These reports described a total of 270 families in which more than one relative had HTLV-1-associated diseases. In most families, different family members suffered from the same disease (*n* = 223). The diseases most frequently reported were ATLL (115 families) and HAM/TSP (102 families). Most families (*n* = 144) included two to four affected individuals. The proportion of ATLL patients with family history of ATLL ranged from 2 to 26%. The proportion of HAM/TSP patients with family history of HAM/TSP ranged from 1 to 48%. The predominant cluster types for ATLL were clusters of siblings and parent-child pairs and for HAM/TSP, an affected parent with one or more affected children. The evidence in the literature, although weak, does suggest that HTLV-1-associated diseases sometimes cluster in families. Whether familial transmission of HTLV-1 is the only determining factor, or whether other factors are also involved, needs further research.

## Introduction

Human T-lymphotropic virus 1 (HTLV-1) is a retrovirus that causes a lifelong infection. HTLV-1 infects an estimated five to ten million people worldwide (Gessain and Cassar, 2012), heterogeneously distributed over all continents. Hyperendemic foci (population prevalence of more than 5%) have been identified in Japan, Australo-Melanesia, the Caribbean, South America, and Central and West Africa. Up to 10% of the people infected with HTLV-1 develop associated diseases (Verdonck et al., 2007b), including (1) inflammatory diseases such as HTLV-1-associated myelopathy/tropical spastic paraparesis (HAM/TSP), HTLV-1-associated uveitis, Sjögren's syndrome, arthropathy, myopathy, and alveolitis; (2) a neoplasm, i.e., adult T-cell leukemia/lymphoma (ATLL); and (3) other infectious complications such as scabies, strongyloidiasis, and tuberculosis. Despite the frequency of the infection and the severity of the associated diseases, HTLV-1 remains a neglected health problem and many questions remain unsolved.

As most of the people infected with HTLV-1 remain asymptomatic, carrying the virus cannot be the only cause of HTLV-1-associated diseases. Some studies have linked specific virus strains with an increased risk of HAM/TSP and ATLL (Furukawa et al., 2000). Nevertheless, it is unlikely that virus genotype plays a major role in the pathogenesis because HTLV-1 is a genetically stable virus with little sequence variation and because there are reports of individuals with the same virus strain but with very different clinical outcomes (Daenke et al., 1990; Van Dooren et al., 2004). On the other hand, there is strong evidence that the proviral load, i.e., the proportion of peripheral blood mononuclear cells that carry the HTLV-1 provirus, is associated with the presence of complications (Nagai et al., 1998). This proviral load depends on the strength of an individual's cytotoxic T-lymphocyte response to HTLV-1, which is associated with the genetically determined human leucocyte antigen (HLA) class 1 types. Associations have indeed been found between specific HLA types and the proviral load (e.g., HLA-A^*^02), HAM/TSP (e.g., HLA-DRB1^*^0101), and ATLL (e.g., HLA-A^*^26) (Jeffery et al., 1999; Sonoda et al., 2011; Assone et al., 2016). These associations, however, could not be replicated across populations (Talledo et al., 2010). Other human genes that could play a role in the pathogenesis include those of nuclear factor kappa B and natural-killer group 2 member D (Talledo et al., 2012). In addition to viral and human genetic factors, there is evidence that the route of HTLV-1 transmission and the duration of exposure may influence the outcome of HTLV-1 infection (Murphy et al., 1989; Maloney et al., 1998). Finally, environmental factors and exposure to co-infections could also play a role (Leon-S and Zaninovic, 1995; Plumelle et al., 1997; LaGrenade et al., 1998). However, in spite of the advances in the understanding of the pathogenesis of HAM/TSP and ATLL, it is still unclear why some individuals develop complications while others do not.

HTLV-1 can be transmitted via contaminated blood products, organ transplantation, sexual intercourse, and from mother to child mainly through breastfeeding. The latter two routes of transmission explain the clustering of HTLV-1 infection in families. Whether familial clustering of HTLV-1-associated diseases can be explained by familial transmission only or whether there are additional factors involved remains a matter of debate. ATLL and HAM/TSP were originally described as sporadic entities. However, soon after the initial characterization of these diseases, reports of families in which several members had ATLL or HAM/TSP started to appear. One possibility is that HTLV-1-associated diseases are distributed randomly in the population of HTLV-1 carriers and that in a few families, more than one case of disease occurs due to chance. However, it is also possible that members of the same family share viral, genetic or environmental factors that increase the risk of associated diseases. Family aggregation studies have proven to be a useful component of the research into diseases such as multiple sclerosis (Gourraud et al., 2011), autoimmune diseases (Cárdenas-Roldán et al., 2013), and cancer (Kiciński et al., 2011; Wan et al., 2015). Evidence from such studies can give new insights in disease mechanisms and improve the quality of diagnosis and counseling.

To explore what is known about family aggregation of HTLV-1-associated diseases, we undertook a systematic review. We aimed at answering if, where and when family aggregation of different HTLV-1-associated diseases had been reported, which relatives were affected and which hypotheses had been proposed to explain the family aggregation.

## Methods

To obtain published information about family aggregation of HTLV-1-associated diseases, we searched MEDLINE (through PubMed), abstract books of HTLV conferences and reference lists of selected papers. To retrieve the abstracts, we hand-searched the abstract books of eleven conferences on HTLV and related viruses organized between 1994 and 2015.

The PubMed search was done in January 2015 and combined three types of search terms: terms indicating (1) HTLV-1 infection, (2) HTLV-1-associated diseases, and (3) family studies. The detailed search strategy is given as Supplementary Material. The search was not restricted by publication date, language, or study design.

Family aggregation of HTLV-1-associated diseases was defined as the occurrence of an HTLV-1-associated disease in more than one member of a family. We used an extensive definition of a family, including in-laws as well as blood relatives. For the literature search, the following conditions were considered to be HTLV-1-associated diseases: HAM/TSP, ATLL, HTLV-1-associated uveitis, and infective dermatitis.

Two reviewers (CA and KV) independently screened all titles and abstracts of the records retrieved through the PubMed search. One reviewer (CA) then read the full text of the preselected records. Doubts and discrepancies in the selection of records were solved through the discussion among two reviewers (CA and KV). All records that mentioned HTLV-1-associated diseases in more than one member of the same family were included. When the same families were identified in more than one record, we selected the record that contained more information and excluded the other. When both records contained the same amount of information, we selected the earliest report.

Data were extracted using a pre-designed form. The following information was extracted from the selected records: study design, year, country, number of families, number of relatives affected by HTLV-1-associated diseases, relationship between affected relatives, and hypotheses proposed to explain family aggregation of HTLV-1-associated diseases.

For the analysis, we first assessed the characteristics of the selected records, including study design. Next, we combined all those studies that gave detailed information about concrete families in which more than one person had an HTLV-1-associated disease. We presented this information as the number of families with particular diseases, particular family relationships or both. Finally, for those studies that were designed to describe or explain family aggregation, we summarized the results of the individual records.

From planning to reporting this review, the recommendations of the PRISMA statement (Preferred Reporting Items for Systematic Reviews and Meta-Analysis; Liberati et al., 2009) were taken into account. However, not all PRISMA items could be followed because they are about intervention studies, which are not the focus of the present review. We did not formally assess the risk of bias but instead, described the study design for all the included records. No protocol was registered for this review and a meta-analysis was not done.

## Results

### Study selection

**Figure 1:**
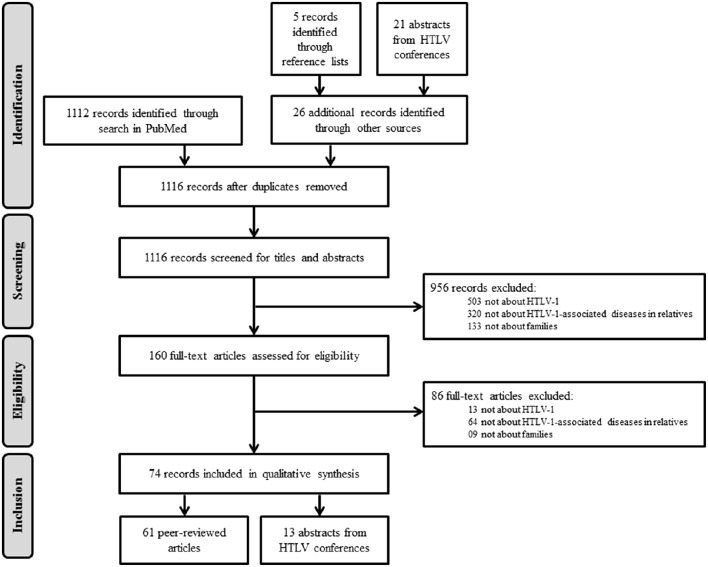
**Flow diagram summarizing systematic search and study selection**.

The MEDLINE search retrieved 1112 records (Figure 1). Reviewing the reference lists of the selected articles yielded five additional records and reviewing 2614 abstracts from HTLV conferences yielded 21 additional records. After removal of duplicate publications, 1116 records passed on to the screening phase. After examination of titles and abstracts, 956 records were excluded because of the following reasons: (1) not about HTLV-1; (2) not focusing on HTLV-1-associated diseases; and (3) not reporting information in families. For the remaining 160 records, we assessed the eligibility of the full-text articles and excluded an additional 86 records based on the same criteria. Figure 1 illustrates the selection process. We finally selected 74 records (61 peer-reviewed articles and 13 abstracts from HTLV conferences) for further analysis.

### Study characteristics

Between 1982 and 2015, 74 studies reported family aggregation of HTLV-1-associated diseases. Thirteen of them were designed to identify or explain family aggregation, including 10 cross-sectional studies (Kondo et al., 1985; Matsuo et al., 1989; Kayembe et al., 1990; Tajima, 1990; Bhigjee et al., 1995; Iwanaga et al., 1995; Pombo-de-Oliveira et al., 2001; Cabada et al., 2007; Alvarez et al., 2014; Nozuma et al., 2014), two reviews (Manns and Qasba, 1999; Shoeibi et al., 2013), and one cohort study (Iwanaga et al., 2010). The remaining 61 records were either case reports or reports of family clusters that were identified within other epidemiological studies. The study design of the records included in this review is summarized in Table 1.

**Table 1:** **Design of the included studies**.

**Study design**	**Number of records**	**References**
Case report	56	
Of one family	46	Imamura et al., 1982; Miyoshi et al., 1982; Kikuchi et al., 1983; Sarin et al., 1983; Kawano et al., 1984; Miyamoto et al., 1985; Taguchi et al., 1985; Yamaguchi et al., 1985; Maekawa et al., 1986; Denic et al., 1988; Ratner and Poiesz, 1988; Sakuma et al., 1988; McKhann et al., 1989; Sanada et al., 1989; Shoji et al., 1989; Denic et al., 1990; Dixon et al., 1990; Nightingale and Desselberger, 1990; Nomura et al., 1990; Ratner et al., 1990; Salazar-Grueso et al., 1990; Uozumi et al., 1991; Cavalcanti et al., 1993; Major et al., 1993; Wilks et al., 1993; Hokezu et al., 1994; Matutes et al., 1995; LaGrenade et al., 1996; Cordoliani et al., 1998; Hu et al., 1998; Gonçalves et al., 1999; Shimizu, 1999; Nakane et al., 2000; Prates et al., 2000; Wilks et al., 2001; Araújo et al., 2002; Biglione et al., 2003; Ribas et al., 2003; Sawa et al., 2005; Nobre et al., 2006; Nomura et al., 2006; Cloves et al., 2009; Daisley and Charles, 2009; Dosik and Wilson, 2009; Suite et al., 2009; Alvarez et al., 2011
Of more than one family	10	Ichimaru et al., 1986; Miyai et al., 1987; Mori et al., 1988; Matsuo et al., 1989; Araki et al., 1993; Blank et al., 1993; Plumelle et al., 1993; Cartier et al., 1998; Nagashima et al., 2001; da Silva et al., 2013
Cross-sectional study	15	Kondo et al., 1985; Mowbray et al., 1989; Kayembe et al., 1990; Tajima, 1990; Bhigjee et al., 1995; Carvalho et al., 1995; Iwanaga et al., 1995; Pombo-de-Oliveira et al., 2001; Furukawa et al., 2003; Mahé et al., 2004; Primo et al., 2005; Cabada et al., 2007; Díaz Torres et al., 2010; Alvarez et al., 2014; Nozuma et al., 2014
Cohort study	1	Iwanaga et al., 2010
Review	2	Manns and Qasba, 1999; Shoeibi et al., 2013

More than half of the reports came from HTLV-1-endemic countries, mainly Japan (*n* = 30) and Brazil (*n* = 10). Reports from non-endemic countries (*n* = 17) described HTLV-1-associated diseases in migrants from endemic regions or in specific ethnic groups (Denic et al., 1988, 1990; Ratner and Poiesz, 1988; Mowbray et al., 1989; Dixon et al., 1990; Nightingale and Desselberger, 1990; Nomura et al., 1990; Ratner et al., 1990; Salazar-Grueso et al., 1990; Major et al., 1993; Matutes et al., 1995; Hu et al., 1998; Prates et al., 2000; Biglione et al., 2003; Mahé et al., 2004; Dosik and Wilson, 2009; Díaz Torres et al., 2010). Table 2 summarizes the countries where family aggregation of HTLV-1-associated diseases has been reported.

**Table 2:** **Countries in which family aggregation of HTLV-1-associated diseases has been reported**.

**Region**	**Country**	Total population^*^	Estimated population infected with HTLV-1^**^	**Number of records reporting family aggregation**	**References**
Asia	Iran	78,868,711	10,000–40,000 (in Mashhad region only)	1	Shoeibi et al., 2013^§^
	Iraq	31,129,225		1	Denic et al., 1990
	Japan	127,368,088	1,080,000–1,300,000	30	Imamura et al., 1982; Miyoshi et al., 1982; Kikuchi et al., 1983; Sarin et al., 1983; Kawano et al., 1984; Kondo et al., 1985; Miyamoto et al., 1985; Taguchi et al., 1985; Yamaguchi et al., 1985; Ichimaru et al., 1986; Maekawa et al., 1986; Miyai et al., 1987; Mori et al., 1988; Sakuma et al., 1988; Matsuo et al., 1989; Sanada et al., 1989; Shoji et al., 1989; Tajima, 1990; Uozumi et al., 1991; Araki et al., 1993; Hokezu et al., 1994; Shimizu, 1999; Nakane et al., 2000; Nagashima et al., 2001; Furukawa et al., 2003; Sawa et al., 2005; Nomura et al., 2006; Iwanaga et al., 1995, 2010; Nozuma et al., 2014
	Taiwan	23,113,901	10,000–30,000	1	Hu et al., 1998
Oceania	Hawaii	1,431,603^***^		2	Dixon et al., 1990; Nomura et al., 1990
North America	United States	313,847,465	90,000–100,000	4	Denic et al., 1988; Ratner and Poiesz, 1988; Ratner et al., 1990; Dosik and Wilson, 2009
The Caribbean	Cuba	11,075,244		1	Díaz Torres et al., 2010
	Guadeloupe	403,314^†^	3000–6000	1	Cordoliani et al., 1998
	Jamaica	2,889,187	100,000–140,000	3	Wilks et al., 1993; LaGrenade et al., 1996; Wilks et al., 2001
	Martinique	388,364^†^	3000–6000	1	Plumelle et al., 1993
	Trinidad and Tobago	1,226,383	9000–18,000	3	Matutes et al., 1995^‡^; Daisley and Charles, 2009; Suite et al., 2009
South America	Argentina	42,192,494		2	Prates et al., 2000; Biglione et al., 2003
	Brazil	205,716,890	300,000–600,000	10	Cavalcanti et al., 1993; Carvalho et al., 1995; Gonçalves et al., 1999; Pombo-de-Oliveira et al., 2001; Araújo et al., 2002; Ribas et al., 2003; Primo et al., 2005; Nobre et al., 2006; Cloves et al., 2009; da Silva et al., 2013
	Chile	17,067,369	90,000–250,000	1	Cartier et al., 1998
	Colombia	45,239,079	1000–1500 (in Tumaco region only)	2	McKhann et al., 1989; Blank et al., 1993
	Paraguay	6,541,591		1	Salazar-Grueso et al., 1990
	Peru	29,549,517	150,000–450,000	3	Cabada et al., 2007; Alvarez et al., 2011, 2014
Europe	United Kingdom	63,047,162	20,000–30,000	4	Mowbray et al., 1989; Nightingale and Desselberger, 1990; Major et al., 1993; Matutes et al., 1995^‡^
Africa	Senegal	12,969,606	30,000–105,000	1	Mahé et al., 2004
	South Africa	48,810,427	180,000–540,000	1	Bhigjee et al., 1995
	Democratic Republic of the Congo	73,599,190	600,000–1,300,000	1	Kayembe et al., 1990
No specific country		–	–	1	Manns and Qasba, 1999^§^

* *Estimates from (The World Factbook, 2012) (www.cia.gov/publications/the-world-factbook)*.

** *Range of the number of people estimated to be infected with HTLV-1 according to Gessain and Cassar (2012)*.

*** *Estimates from The U.S. Census Bureau. Census (2015) (http://www.census.gov)*.

† *According to the estimates of the Institut national de la statistique et des études économiques, France, 2012 (www.insee.fr) [Institut national de la statistique et des études économiques. Territoire. Régions, departements et villes de France. Département de La Guadeloupe. Available online at: www.insee.fr/fr/themes/comparateur.asp?codgeo=dep-971 (Accesed March 4, 2016); Institut national de la statistique et des études économiques. Territoire. Régions, departements et villes de France. Département de La Martinique. Available online at: www.insee.fr/fr/themes/comparateur.asp?codgeo=dep-972 (Accesed March 4, 2016)]*.

‡ *Matutes et al. describe a family that includes residents in Trinidad and Tobago, migrants from Trinidad and Tobago to the United Kingdom and members of the same family born in the United Kingdom (Matutes et al., 1995)*.

§ *Review article*.

### Overview of families

Overall, there were 270 families in which more than one family member had an HTLV-1-associated disease (Table 3). In 223 of these families (83%), several family members suffered from the same disease, i.e., ATLL (115 families), HAM/TSP (102 families), or another disease (6 families). In 47 families (17%), different family members suffered from different diseases. Some of the included records also described the coexistence of more than one HTLV-1-associated disease in the same person (Supplementary Table).

**Table 3:** **Overview of family clusters reported in the literature: number of records, families, and affected persons according to type of relative and type of disease**.

**Type of relatives**	**Number of HTLV-1-associated diseases**	**Disease**	**Number of records**	**Number of families**	**Number of persons/number of families**	**References**
Blood relatives	One disease	ATLL	22	44	102 persons/44 families	Imamura et al., 1982; Miyoshi et al., 1982; Kikuchi et al., 1983; Sarin et al., 1983; Kawano et al., 1984; Kondo et al., 1985; Miyamoto et al., 1985; Taguchi et al., 1985; Yamaguchi et al., 1985; Ichimaru et al., 1986; Maekawa et al., 1986; Denic et al., 1988; Ratner and Poiesz, 1988; Nomura et al., 1990; Ratner et al., 1990; Wilks et al., 1993; Iwanaga et al., 1995; Matutes et al., 1995; Cordoliani et al., 1998; Shimizu, 1999; Nomura et al., 2006; Dosik and Wilson, 2009
		HAM/TSP	11	26	63 persons/25 families	Miyai et al., 1987; Mori et al., 1988; Dixon et al., 1990; Kayembe et al., 1990; Salazar-Grueso et al., 1990; Carvalho et al., 1995; Cartier et al., 1998; Nakane et al., 2000; Biglione et al., 2003; Ribas et al., 2003; Primo et al., 2005
		Infective dermatitis	2	2	5 persons/2 families	Suite et al., 2009; da Silva et al., 2013
		Uveitis	1	2	4 persons/2 families	Araki et al., 1993
	More than one disease	HAM/TSP and ATLL	8	12	27 persons/12 families	Denic et al., 1990; Tajima, 1990; Uozumi et al., 1991; Blank et al., 1993; Hu et al., 1998; Prates et al., 2000; Pombo-de-Oliveira et al., 2001; Díaz Torres et al., 2010
		HAM/TSP and infective dermatitis	5	17	44 persons/17 families	LaGrenade et al., 1996; Gonçalves et al., 1999; Araújo et al., 2002; Primo et al., 2005; da Silva et al., 2013
		HAM/TSP and myositis	1	1	2 persons/1 family	Hokezu et al., 1994
		ATLL and uveitis	1	1	2 persons/1 family	Pombo-de-Oliveira et al., 2001
		ATLL and strongyloidiasis	1	1	2 persons/1 family	Alvarez et al., 2011
		HAM/TSP, ATLL and infective dermatitis	1	1	3 persons/1 family	Wilks et al., 2001
		HAM/TSP, ATLL, and strongyloidiasis	1	1	2 persons/1 family	Blank et al., 1993
		Uveitis, keratoconjuntivitis, polyneuropathy, and lymphoma	1	1	5 persons/1 family	Sawa et al., 2005
		Infective dermatitis, scabies, and other neurological signs	1	1	6 persons/1 family	Mahé et al., 2004
In-laws	One disease	ATLL	3	3	6 persons/3 families	Sakuma et al., 1988; Sanada et al., 1989; Pombo-de-Oliveira et al., 2001
	More than one disease	HAM/TSP and ATLL	3	3	6 persons/3 families	Mowbray et al., 1989; Nightingale and Desselberger, 1990; Major et al., 1993
Blood relatives and in-laws	One disease	HAM/TSP	2	2	7 persons/2 families	McKhann et al., 1989; Cavalcanti et al., 1993
	More than one disease	HAM/TSP and infective dermatitis	1	2	6 persons/2 families	da Silva et al., 2013
		HAM/TSP and other dermatological conditions	1	1	3 persons/1 family	Nobre et al., 2006
Not specified		ATLL	5	68		Tajima, 1990; Pombo-de-Oliveira et al., 2001; Furukawa et al., 2003; Cabada et al., 2007; Iwanaga et al., 2010
		HAM/TSP	4	73	59 persons/26 families	Matsuo et al., 1989; Bhigjee et al., 1995; Alvarez et al., 2014; Nozuma et al., 2014
		HAM/TSP and ATLL	2	5	3 persons/1 family	Plumelle et al., 1993; Daisley and Charles, 2009
		Polyneuropathy	1	2	5 persons/2 families	Nagashima et al., 2001
		Dermatological disorders	1	1		Cloves et al., 2009

*ATLL, adult T-cell leukemia/lymphoma; HAM/TSP, HTLV-1-associated myelopathy/tropical spastic paraparesis*.

The number of affected individuals per family ranged from two to seven. Most of the family clusters (*n* = 144) contained two to four affected individuals. However, we also found reports of more complex families in which there were three or more different associated diseases (5 families), five or more affected individuals (6 families), and/or affected individuals in three or more generations (3 families).

Family aggregation has been reported in blood relatives and in in-laws. The type of family relationship between the cases was given for 121 out of the 270 families included in this review (45%). In 110 families, the diseases affected only blood relatives, in six families only in-laws, and in five families, the diseases affected both blood relatives and in-laws. In the 110 families in which only blood relatives were affected, the diseases that clustered most frequently were ATLL (44 families), HAM/TSP (27 families), HAM/TSP + infective dermatitis (17 families), and HAM/TSP + ATLL (13 families). The six families in which only in-laws developed diseases comprised three pairs of spouses with ATLL and three pairs of spouses in which the husband had ATLL and the wife had HAM/TSP (Table 3).

Detailed relationships between the cases were given in 51 family clusters of ATLL, 47 clusters of HAM/TSP, and 17 of ATLL + HAM/TSP (Table 4). For ATLL, the most frequent pattern was a cluster of two or more affected siblings (26 out of 51 clusters, 51%). Parent-child pairs with ATLL were also relatively common (11 of 51 clusters, 22%). For HAM/TSP, the predominant cluster type was that of one affected parent with one or more affected children (29 of 47 clusters, 62%). In families with ATLL + HAM/TSP, most of the cases corresponded to parent-child pairs (6 of 17 clusters, 35%) or sibling pairs (5 of 17 clusters, 29%).

**Table 4:** **Relationship between patients with familial adult T-cell leukemia/lymphoma or familial HTLV-1-associated myelopathy/tropical spastic paraparesis**.

	**ATLL**		**HAM/TSP**		**ATLL and HAM/TSP**	
**Relation between affected subjects**	**Number of clusters**	**References**	**Number of clusters**	**References**	**Number of clusters**	**References**
One parent and one child	11	Sarin et al., 1983; Kondo et al., 1985; Ichimaru et al., 1986; Denic et al., 1988; Dosik and Wilson, 2009; Alvarez et al., 2011	19	Miyai et al., 1987; Mori et al., 1988; Denic et al., 1990; Kayembe et al., 1990; LaGrenade et al., 1996; Cartier et al., 1998; Araújo et al., 2002; Primo et al., 2005; da Silva et al., 2013	6	Shoji et al., 1989; Denic et al., 1990; Blank et al., 1993; Hu et al., 1998; Wilks et al., 2001
One parent and two or more children	1	Iwanaga et al., 1995	10	Mori et al., 1988; Kayembe et al., 1990; Carvalho et al., 1995; Primo et al., 2005; da Silva et al., 2013	1	Prates et al., 2000
Two siblings	23	Imamura et al., 1982; Miyoshi et al., 1982; Kawano et al., 1984; Kondo et al., 1985; Miyamoto et al., 1985; Taguchi et al., 1985; Ichimaru et al., 1986; Maekawa et al., 1986; Ratner and Poiesz, 1988; Nomura et al., 1990; Ratner et al., 1990; Plumelle et al., 1993; Wilks et al., 1993; Iwanaga et al., 1995; Matutes et al., 1995; Cordoliani et al., 1998; Daisley and Charles, 2009	8	Miyai et al., 1987; Dixon et al., 1990; Hokezu et al., 1994; Cartier et al., 1998; Nakane et al., 2000; Biglione et al., 2003; Ribas et al., 2003; Primo et al., 2005	5	Pombo-de-Oliveira et al., 2001
More than two siblings	3	Yamaguchi et al., 1985; Ichimaru et al., 1986; Nomura et al., 2006	–	–	–	–
Two siblings and the child of one of them	5	Kikuchi et al., 1983; Ichimaru et al., 1986; Shimizu, 1999	–	–	1	Uozumi et al., 1991
Two spouses	3	Sakuma et al., 1988; Sanada et al., 1989; Pombo-de-Oliveira et al., 2001	1	da Silva et al., 2013	3	Mowbray et al., 1989; Nightingale and Desselberger, 1990; Major et al., 1993
Two spouses and one child	–	–	2	McKhann et al., 1989; da Silva et al., 2013	–	–
Other	5	Kondo et al., 1985; Pombo-de-Oliveira et al., 2001	7	Kayembe et al., 1990; Salazar-Grueso et al., 1990; Cavalcanti et al., 1993; Nobre et al., 2006	1	Díaz Torres et al., 2010

*ATLL, adult T-cell leukemia/lymphoma; HAM/TSP, HTLV-1-associated myelopathy/tropical spastic paraparesis. The same family was counted only once; some references describe several families*.

### Family aggregation of HTLV-1-associated diseases

Thirteen studies contained elements in their design that allowed to describe the family aggregation of HTLV-1-associated diseases in a systematic way. Nine of these studies reported which proportion of patients with ATLL or HAM/TSP had at least one relative with the same disease (Table 5). The proportion of ATLL patients with a family history of ATLL ranged from 2 to 26%. The proportion of HAM/TSP patients with a family history of HAM/TSP ranged from 1 to 48% (Table 5).

**Table 5:** **Proportion of patients with HTLV-1-associated diseases who have a relative with the same disease**.

**Disease**	**Country**	**Number of cases**	**Number (proportion) of cases who have at least one relative with the same disease**	**References**
ATLL	Japan	657	14 (2%)	Tajima, 1990
ATLL	Japan	23	2 (9%)	Iwanaga et al., 1995
ATLL	Japan	38	9 (24%)	Kondo et al., 1985
ATLL	Brazil	82	3 (4%)	Pombo-de-Oliveira et al., 2001
ATLL	Peru	42	11 (26%)	Cabada et al., 2007
HAM/TSP	Japan	21	6 (29%)	Matsuo et al., 1989
HAM/TSP	South Africa	124	1 (1%)	Bhigjee et al., 1995
HAM/TSP	DRC	21	10 (48%)	Kayembe et al., 1990
HAM/TSP	Iran	NA	NA (9–25%)	Shoeibi et al., 2013

*ATLL, adult T-cell leukemia/lymphoma; HAM/TSP, HTLV-1-associated myelopathy/tropical spastic paraparesis; NA: not available*.

The remaining four aggregation studies used different approaches. Iwanaga et al. conducted a large cohort study in Japan. They analyzed the information of 1218 HTLV-1-infected subjects who did not have HAM/TSP or ATLL at the beginning of the study. The majority of the study participants (65%) were women; 55% were born in Southern Japan and the median age at enrollment was 60 years for women and 58 years for men. During follow up, 14 study participants developed ATLL. The incidence of ATLL in this cohort was 7 per 1000 person-years. On multivariable Cox regression analysis, four factors were significantly associated with an increased hazard of developing ATLL: high baseline proviral load, advanced age, first diagnosis of HTLV-1 infection during treatment for other diseases, and family history of ATLL. After adjustment for other associated factors, the hazard of developing ATLL was 12 times higher in those HTLV-1 carriers who had a family history of ATLL compared to those who did not have such a family history (hazard ratio 12.1; 95% confidence interval 2.3–64.7) (Iwanaga et al., 2010).

Alvarez et al. set out to evaluate if having a relative with HAM/TSP increases the risk of having HAM/TSP. They expected that the frequency of HAM/TSP among relatives of HAM/TSP patients would be higher than that among relatives of asymptomatic HTLV-1 carriers. In a study in Peru, they found 30 HAM/TSP cases (9%) among 318 HTLV-1-positive relatives of 334 HAM/TSP patients compared to 15 HAM/TSP cases (7%) among 204 HTLV-1-positive relatives of 230 asymptomatic HTLV-1 carriers. This difference was not statistically significant. The authors concluded that HAM/TSP is usually sporadic but that in some particular families there are HAM/TSP clusters (Alvarez et al., 2014).

Manns et al. started from the hypothesis that human genetic characteristics are a causal factor for HTLV-1-associated diseases. They reviewed the literature to check if the patterns of disease aggregation in families supported this hypothesis. They identified 19 families with multiple cases of ATLL and 16 families with multiple cases of HAM/TSP. The authors concluded that the patterns of disease aggregation differed between ATLL and HAM/TSP families, but they did not find strong arguments in favor of a genetic basis for the aggregation (Manns and Qasba, 1999).

Nozuma et al. conducted a study in Japan in which they compared clinical and laboratory characteristics of 124 sporadic HAM/TSP cases with those of 40 HAM/TSP patients with a family history of HAM/TSP. The HAM/TSP cases with a family history of HAM/TSP had a slower rate of HAM/TSP progression and an earlier age of onset (mean 41.3 years) than sporadic cases (mean 51.6 years). There was no difference in HTLV-1 proviral load between the two groups (Nozuma et al., 2014). Four other records mention an early age of onset of HAM/TSP in some relatives of HAM/TSP patients (Miyai et al., 1987; McKhann et al., 1989; Kayembe et al., 1990; Cartier et al., 1998), but only Nozuma et al. evaluated this in a systematic way (Nozuma et al., 2014).

### Hypotheses to explain family aggregation

Diverse hypotheses were proposed in the included papers to explain family aggregation of HTLV-1-associated diseases. Clustering of cases in families was attributed to viral genetic factors, host genetic and immune factors, transmission routes, environmental factors or a combination of these (Table 6).

**Table 6:** **Hypotheses proposed to explain family aggregation of HTLV-1-associated diseases**.

**Factor**		**Hypothesis**	References^*^
Virus genetics		Particular retroviral strains, specific virus subgroups/mutations/deletions	Ichimaru et al., 1986; Cavalcanti et al., 1993; Major et al., 1993; Nakane et al., 2000; Mahé et al., 2004; Nomura et al., 2006
		Viral strains specific for ATLL	Maekawa et al., 1986; Nomura et al., 1990; Ratner et al., 1990
		Neurotropic viral strains	Miyai et al., 1987; Kayembe et al., 1990; Salazar-Grueso et al., 1990
Host	Genetic and/or Immune factors	Specific HLA alleles	Shoji et al., 1989; Uozumi et al., 1991; Blank et al., 1993; LaGrenade et al., 1996; Nakane et al., 2000; Pombo-de-Oliveira et al., 2001; Primo et al., 2005; Sawa et al., 2005; Nomura et al., 2006
		Genetic susceptibility for disease (not specified)	Imamura et al., 1982; Miyai et al., 1987; Kayembe et al., 1990; Salazar-Grueso et al., 1990; Araki et al., 1993; Cavalcanti et al., 1993; Wilks et al., 1993; Cartier et al., 1998; Cordoliani et al., 1998; Wilks et al., 2001; Mahé et al., 2004; Dosik and Wilson, 2009; Alvarez et al., 2011
		Different immunogenetic backgrounds leading to different disease outcomes	Shoji et al., 1989
		Specific immune characteristics in patients	Wilks et al., 1993; Hokezu et al., 1994; Díaz Torres et al., 2010
		Exacerbated humoral response	Sarin et al., 1983; Ratner and Poiesz, 1988; Cartier et al., 1998; Pombo-de-Oliveira et al., 2001; Wilks et al., 2001; Primo et al., 2005; Cloves et al., 2009; Nozuma et al., 2014
		Particular integration sites	Salazar-Grueso et al., 1990
	Transmission routes	Vertical (milk-borne) transmission	Mori et al., 1988; Matsuo et al., 1989
		High provirus load in breastmilk, fluctuating viremia in mother	Salazar-Grueso et al., 1990; da Silva et al., 2013
		Infection in childhood, long incubation period	Ichimaru et al., 1986; Maekawa et al., 1986; Wilks et al., 1993
		Not specified	Manns and Qasba, 1999
Environment		Not specified	Kayembe et al., 1990; Cavalcanti et al., 1993; Mahé et al., 2004; Dosik and Wilson, 2009; Alvarez et al., 2011

*ATLL, adult T-cell leukemia/lymphoma; HLA, human leukocyte antigen*.

* *Some studies propose more than one hypothesis*.

With regard to the virus, some authors thought of specific strains causing either ATLL or HAM/TSP. Mahé et al. suggested that there were specific point mutations or “familial signatures” in family clusters of infective dermatitis (Mahé et al., 2004). Along the same line, Renjifo et al. suggested specific *rex* and *env* mutations in a cluster of three HAM/TSP patients (two parents and their child; Renjifo et al., 1995). On the other hand, there was also a report of a family with different clinical outcomes (father with ATLL, mother with HAM/TSP, and three asymptomatic HTLV-1-infected children) that could not be explained by different viral strains. In this family, Major et al. found a complete sequence conservation of the *tax* gene (Major et al., 1993).

At the level of the human host, many authors mentioned the possible causal role of genetic, and immunological factors. In 14 records, it was specified what these factors could be, e.g., specific HLA alleles, or an exacerbated humoral response. One study checked HLA haplotypes in a family with infective dermatitis and HAM/TSP (LaGrenade et al., 1996). They found that a mother with infective dermatitis + HAM/TSP and her two children, a son with infective dermatitis + pyramidal tract involvement and an asymptomatic son, shared the same HLA alleles while their other asymptomatic HTLV-1-infected relatives presented other HLAs. Furthermore, the HLAs shared by the mother and her children had been linked to HAM/TSP in Japanese patients (Usuku et al., 1988). Similarly, Nomura et al. found that two siblings coming from a family in which six siblings presented ATLL shared the same HLA alleles, which had been proposed to predispose to ATLL (Yashiki et al., 2001; Nomura et al., 2006).

Nakane et al. explored both the HTLV-1 sequences and the HLA genotypes and their role in the clinical outcome of HTLV-1. In a family of four HTLV-1 carriers (an asymptomatic mother, two brothers with HAM/TSP—one of them a twin—and an asymptomatic twin), they found that the monozygotic twins with different clinical outcomes carried different viral strains. They also found that different HLA molecules were expressed in the mother and her children (Nakane et al., 2000).

Information about the proviral load was given in four records, but different methods (based on *tax* gene, *pX* gene, or whole genome) were used to measure this (Wilks et al., 2001; Furukawa et al., 2003; Iwanaga et al., 2010; Nozuma et al., 2014). In two studies, asymptomatic HTLV-1-carriers with a family history of ATLL or HAM/TSP were found to have a higher proviral load than those without such a family history (Furukawa et al., 2003; Iwanaga et al., 2010).

The route of HTLV-1 transmission was also put forward as a factor that could influence the outcome of infection, through the infective dose (provirus load in breastmilk) or the timing of HTLV-1 infection (during childhood vs. during adulthood; Ichimaru et al., 1986; Mori et al., 1988; Matsuo et al., 1989; Salazar-Grueso et al., 1990; Wilks et al., 1993; da Silva et al., 2013). None of the included studies explored this further. Finally, five records mentioned that environmental factors could play a role but this was not further specified or studied (Table 6).

## Discussion

The occurrence of several family members affected by HTLV-1-associated diseases has been reported ever since the discovery of HTLV-1 in the early 1980s (Imamura et al., 1982; Miyoshi et al., 1982). Descriptions of family clusters come from all continents. Known HTLV-1-endemic regions such as Japan and Brazil and places with many immigrants from HTLV-1-endemic regions such as the United Kingdom are particularly well-represented among the reports. However, there are also other HTLV-1-endemic regions such as Romania for which we did not find any reports of family aggregation (Laperche et al., 2009; European Centre for Disease Prevention Control, 2015). Although our search retrieved many records (*n* = 74), few studies were specifically designed to investigate family aggregation. Nonetheless, we found descriptions of 270 concrete families in which more than one person had an HTLV-1-associated disease. The specific diseases and the relationships within the affected families varied, but the predominant situation was that several blood relatives suffered from the same HTLV-1-associated disease. The majority of the reports were about ATLL, HAM/TSP, or both.

An important limitation of this review is that the majority of the included studies are case reports describing one or several families. In addition, some of the included information came from conference abstracts. We decided not to exclude studies based on study design or risk of bias in order to give a broad overview of all the available information. As a consequence, the findings have to be interpreted with caution. A second limitation is that the extent to which the families were studied and the quality of the diagnosis of HTLV-1-associated diseases varied across the included reports. For example, some authors only described cases with a diagnosis based on clear clinical and laboratory arguments whereas others also included cases based on an interview about their relatives. In addition, many authors did not report how they had diagnosed the HTLV-1-associated diseases. As a rule, we accepted the diagnoses as they were described in the included papers, but sometimes it was difficult to decide what to do with diagnoses such as “pre-ATLL” or “non-HAM/TSP neurological disorders.” Furthermore, several asymptomatic HTLV-1-positive individuals could develop HTLV-1-associated diseases later on. Third, there is the issue of publication bias, which may work in two directions. Under-reporting is likely because HTLV-1 infection is a neglected topic which is frequent in some areas without a strong publication record. Many cases of HTLV-1-associated diseases might never be diagnosed, and outside Japan, there are no systematic registries of HTLV-1-associated diseases. On the other hand, over-reporting is also possible because the most extreme or unusual family clusters may have a higher chance of being published. Finally, it is possible that relatives of people with HTLV-1-associated diseases get screened for HTLV-1 more frequently than relatives of asymptomatic HTLV-1 carriers and that, consequently, they get better access to medical care and diagnosis of HTLV-1-associated diseases. If true, this may increase the chance of finding family clusters.

The design and execution of studies on family aggregation of HTLV-1-associated diseases are challenging for many reasons. Such studies require serological and clinical evaluations of many family members, some of whom may not be available or not willing to participate. In addition, in most families, only some individuals are HTLV-1 infected and only those who are infected are at risk of developing complications. The fact that the patterns of infection differ across families adds an extra layer of complexity to the study of family aggregation of HTLV-1-associated diseases, as aggregation has to be investigated on top of the probability to be infected within a family. Finally, as the incubation time of HTLV-1-associated diseases can be very long and there are no markers that predict disease occurrence, robust studies will require large sample sizes, long follow-up periods or both. Such large population-based family studies have been done for other diseases such as multiple sclerosis in Sweden, systemic lupus erythematosus in Taiwan and liver cancer in China, but not yet for HTLV-1 (Hemminki et al., 2009; Kuo et al., 2015; Wan et al., 2015).

The central question of this review was: do HTLV-1-associated diseases run in families? Or phrased differently: does having a relative with an HTLV-1-associated disease increase an HTLV-1 carrier's risk to develop an associated disease as well? The mere number of records about the topic (*n* = 74) as well as the number of reported family clusters (*n* = 270) suggest that HTLV-1-associated diseases do occur in families more frequently than would be expected by chance. Moreover, one cohort study showed that having a family history of ATLL increases the risk of developing ATLL (Iwanaga et al., 2010). This cohort study was comprehensive and well-designed to answer the question of family aggregation and contributes the strongest evidence (Iwanaga et al., 2010). The majority of the other studies that we retrieved were case reports or case series in which the risk of bias is known to be high. Therefore, we considered that the overall strength of the evidence was weak.

Familial predisposition for a disease is sometimes used as a surrogate measure for the interaction between genetic and environmental factors (Nielsen et al., 2015), and in this case also viral factors. The fact that there were many clusters of blood relatives with ATLL or HAM/TSP supports the human genetic component in the causal model of HTLV-1-associated diseases. On the other hand, this human genetic component is clearly not sufficient to explain the development of these diseases, because (1) there are also clusters of in-laws, (2) there are families in which different relatives have different diseases, and (3) the concordance rate of monozygotic twins is < 100% (Nakane et al., 2000; Alvarez et al., 2011). Furthermore, the genetic factors that have been identified so far in association with ATLL or HAM/TSP do not have a very strong effect on disease risk or do not have the same effect in all populations (Vine et al., 2002; Talledo et al., 2010). Therefore, ATLL and HAM/TSP seem to be complex diseases just like among others diabetes, obesity, asthma, multiple sclerosis, and other autoimmune disorders, which depend on the effects of multiple genes in combination with lifestyle and environmental factors. Such complex diseases typically cluster in families, but without a simple pattern of inheritance.

Vertical transmission has been linked to the development of ATLL (Murphy et al., 1989) and horizontal transmission to HAM/TSP (Maloney et al., 1998). Similarly, transmission routes have been proposed to play a role in the family aggregation of HTLV-1-associated diseases. In the case of ATLL, our findings are in line with this hypothesis, as most of the ATLL clusters consisted of siblings who most likely acquired the infection vertically. In the case of HAM/TSP, we had expected to find more clusters of in-laws; instead, the majority of the HAM/TSP clusters were parent-child or sibling pairs. It is important to note in this context that there may be differences between familial and sporadic HAM/TSP: familial HAM/TSP has been reported to start at an earlier age and to be less severe than sporadic HAM/TSP (Nozuma et al., 2014). One way of bringing several causal components together would be to think of HAM/TSP as a disease that occurs in people (1) with a genetic tendency (based on HLA among other genes) to develop inflammatory conditions, (2) carrying an HTLV-1 strain with strong expression of antigenic Tax protein, and (3) infected with HTLV-1 via sexual intercourse (sporadic, late onset HAM/TSP) or via breastfeeding (familial, early onset HAM/TSP). ATLL could then be seen as a disease that occurs in people (1) with genetic susceptibility (based on HLA among other genes) to infections, (2) carrying an HTLV-1 strain with dominant HTLV-1 bZIP factor and weak Tax expression, and (3) infected with HTLV-1 mainly via breastfeeding.

Environmental factors were mentioned several times as a possible and partial explanation of family aggregation of HTLV-1-associated diseases. However, none of the included records explored this further. It is noticeable that co-infections were not mentioned in this context, because co-infections may also run in families and because specific co-infections have been linked to HTLV-1-associated diseases before, particularly infective dermatitis with HAM/TSP (Bittencourt and de Oliveira, 2010), and strongyloidiasis with ATLL (Plumelle et al., 1997). Furthermore, there is evidence that HTLV-1 can influence the outcome and severity of other infections such as tuberculosis (Verdonck et al., 2007a), HIV (Brites et al., 2001; Silva et al., 2009), and hepatitis C (Castro and Roger, 2016). Although none of the records suggested the role of co-infections in explaining family aggregation, they did report families in which co-infections (i.e., strongyloidiasis and infective dermatitis) were present (Blank et al., 1993; Wilks et al., 1993; LaGrenade et al., 1996; Gonçalves et al., 1999; Araújo et al., 2002; Mahé et al., 2004; Primo et al., 2005; Nobre et al., 2006; Suite et al., 2009; Alvarez et al., 2011; da Silva et al., 2013).

The implications of this review for clinical practice relate mainly to counseling. When a person is diagnosed with an HTLV-1-associated disease, a family study is usually done. Frequently asked questions during counseling include: “Will I or my relatives develop the same disease?” and “What can we do to prevent HTLV-1-associated diseases?” Given the limited knowledge of the factors implicated in the development of HTLV-1-associated diseases and, in consequence, the lack of measures to prevent them, it remains difficult to answer such questions. However, we think that based on this review, the possibility of a familial predisposition to HTLV-1-associated diseases should be mentioned during counseling. In addition, close clinical follow up of the HTLV-1-infected relatives of patients with ATLL or HAM/TSP appears to be indicated. Potential biomarkers such as the proviral load (Nagai et al., 1998) and cytokine profiles (Starling et al., 2013) deserve further study as they could make counseling more meaningful.

This review also has implications for research. ATLL and HAM/TSP seem to be complex diseases, which are notably difficult to study. Further research on ATLL and HAM/TSP in families should benefit from important advances in research about other complex diseases in which specific gene-environment interactions are being investigated and for which new methods are being developed (Esposito et al., 2015; Park and Kim, 2015). Family studies could contribute to the research about the causes of HTLV-1-associated diseases, but it is clear that in order to be really useful, future studies will have to be large, well-designed, and hypothesis driven.

In conclusion, families with several cases of HTLV-1-associated diseases have caught the attention of clinicians and researchers in different times and different continents. Although the evidence is weak, it does suggest that HTLV-1-associated diseases sometimes cluster in families.

## Author contributions

CA and KV conceived and designed this systematic review; screened and selected the articles; and drafted the manuscript. CA, KV, EG, and AV analyzed and interpreted the information. KV, EG, and AV critically revised the manuscript. All authors read and approved the final version.

## Funding

The first author (CA) received scholarships from the Belgian Development Cooperation through the Flemish Interuniversity Council (VLIR-UOS) ZEIN2010PR376 and the Consejo Nacional de Ciencia, Tecnología e Innovación Tecnológica (CONCYTEC-CIENCIACTIVA) of the Peruvian Government. This research was supported by VLIR-UOS grant (ZEIN2010PR376) and “Vaast Leysen Leerstoel voor Wetenschappelijk onderzoek over infectieziekten in ontwikkelingslanden” from KU Leuven, Belgium.

### Conflict of interest statement

The authors declare that the research was conducted in the absence of any commercial or financial relationships that could be construed as a potential conflict of interest.
