# Supplementary material for: Family Aggregation of Human T-Lymphotropic Virus 1-Associated Diseases: A Systematic Review
Source: Front Microbiol. 2016 Oct 28;7:1674. doi: 10.3389/fmicb.2016.01674 (PMC5083714; doi:10.3389/fmicb.2016.01674)
Supplement: Supplementary file 1 [file Table1.docx]

***Supplementary Material***

**Family Aggregation of Human T-Lymphotropic Virus 1-Associated Diseases: a Systematic Review**

**Carolina Alvarez*, Eduardo Gotuzzo, Anne-Mieke Vandamme, Kristien Verdonck**

***Correspondence:** Carolina Alvarez: [carolina.alvarez@upch.pe](mailto:carolina.alvarez@upch.pe)

**Supplementary table. Studies reporting more than one HTLV-1-associated disease in a person**

| **HTLV-1-associated diseases** | **Number of cases** | **References** |
| --- | --- | --- |
| HAM/TSP and infective dermatitis | 21 | LaGrenade et al., 1996; Araujo et al., 2002; Primo et al., 2005; da Silva et al., 2013. |
| HAM/TSP and ATLL | 1 | Denic et al., 1990 |
| HAM/TSP and small-cell lymphoma | 1 | Cavalcanti et al., 1993 |
| HAM/TSP and sicca syndrome | 3 | Cartier et al., 1998 |
| HAM/TSP, sicca syndrome and mycosis fungoides | 1 | Cartier et al., 1998 |
| HAM/TSP and mycosis fungoides | 1 | Cartier et al., 1998 |
| HAM/TSP and myositis | 2 | Hokezu et al., 1994 |
| HAM/TSP and multiple skin conditions | 2 | Nobre et al., 2006 |
| ATLL and infective dermatitis* | 1 | Mahé et al., 2004 |
| ATLL and tuberculosis | 1 | Sakuma et al., 1988 |
| ATLL and strongyloidiasis** | 4 | Blank et al., 1993; Alvarez et al., 2011 |
| Infective dermatitis and scabies | 2 | Mahé et al., 2004 |
| Polyneuropathy and uveitis | 1 | Sawa et al., 2005 |
| Polyneuropathy and tuberculosis | 1 | Sawa et al., 2005 |

HAM/TSP: HTLV-1-associated myelopathy/tropical spastic paraparesis

ATLL: adult T-cell leukemia/lymphoma

*Patient with ATLL and infective dermatitis not corresponding to any of the families reported.

** Two patients with ATLL and strongyloidiasis did not belong to families included in this review.
