# Supplementary material for: Family Aggregation of Human T-Lymphotropic Virus 1-Associated Diseases: A Systematic Review
Source: Front Microbiol. 2016 Oct 28;7:1674. doi: 10.3389/fmicb.2016.01674 (PMC5083714; doi:10.3389/fmicb.2016.01674)
Supplement: Supplementary file 2 [file DataSheet1.docx]

***Supplementary Material***

**Family Aggregation of Human T-Lymphotropic Virus 1-Associated Diseases: a Systematic Review**

**Carolina Alvarez*, Eduardo Gotuzzo, Anne-Mieke Vandamme, Kristien Verdonck**

***Correspondence:** Carolina Alvarez: [carolina.alvarez@upch.pe](mailto:carolina.alvarez@upch.pe)

**Supplementary Data. PubMed search strategy**

(((((HTLV) OR (human T-lymphotropic virus) OR (ATLV) OR (Adult T Cell Leukemia Virus) OR (Leukemia Lymphoma Virus) OR (human T-cell lymphotropic virus) OR (human T-cell leukaemia virus)))) AND (((((spastic parapareses) OR (spastic paraparesia) OR (HTLV-1 associated myelopathy) OR (HTLV-I-associated myelopathy) OR (paraplegia, tropical spastic)) OR (Leukemia-Lymphoma, Adult T-Cell) OR (Adult T-Cell Leukemia-Lymphomas) OR (HTLV I Associated T Cell Leukemia Lymphoma) OR (HTLV-Associated Leukemia-Lymphoma) OR (HTLV Associated Leukemia Lymphoma) OR (HTLV-Associated Leukemia-Lymphomas) OR (HTLV-I-Associated T-Cell Leukemia-Lymphomas) OR (Adult T-Cell Leukemias) OR (Adult T-Cell Leukemia) OR (Lymphoma) OR (Leukemia) OR (Leukaemia) OR (ATL) OR (ATLL) ) OR (uveitis) OR (Uveitides)) OR (dermatitis) OR (dermatitides))) AND ((((((((((((((((((((((family) OR familial)) OR relatives)) OR research family)) OR pedigree)) OR genealogical)) OR cluster)) OR clustering)) OR grouping)) OR aggregation)) OR segregation)) OR heritability)
